# Trunk kinematics during seated functional activities in individuals with spinal cord injury: a systematic review and meta-analysis

**DOI:** 10.1038/s41598-025-06765-5

**Published:** 2025-07-01

**Authors:** Maria del Rocio Hidalgo Mas, Ruo-Yan Wu, Tom Nightingale, Eduardo Martinez Valdes, Zubair Ahmed, Shin-Yi Chiou

**Affiliations:** 1 School of Sport, Exercise and Rehabilitation Sciences, University of Birmingham, Edgbaston, Birmingham, B15 2TT UK; 2 The Department of Physical Therapy and Assistive Technology, National Yang Ming Chiao Tung University, Taipei, 112 Taiwan; 3 Centre for Trauma Sciences Research, University of Birmingham, Edgbaston, Birmingham, B15 2TT UK; 4 Department of Inflammation and Ageing, School of Infection, Inflammation and Immunology, College of Medicine and Health, University of Birmingham, Edgbaston, Birmingham, B15 2TT UK; 5 University Hospitals NHS Foundation Trust, Mindelsohn Way, Edgbaston, Birmingham, B15 2TT UK

**Keywords:** Reaching, Transfer, Wheeling, Paraplegia, Tetraplegia, Health occupations, Medical research

## Abstract

This systematic review with meta-analysis compared the differences in trunk kinematics between persons living with spinal cord injury (PwSCI) and non-SCI individuals during sitting-based daily activities. A systematic search was conducted in MEDLINE, EMBASE, CINAHL-PLUS and Web of Science databases to identify relevant studies from inception up until 22nd March 2024. A total of 36 studies with 444 participants (mean age 38.9 ± 8.4 years; 361 males) were included, of whom 272 had AIS A/B classification, with injury levels ranging from C4 to L2. Three main tasks were identified: reaching, transfers, and wheeling. Four studies reported data on trunk displacement and a meta-analysis indicated that healthy controls showed significantly greater trunk displacement than SCI participants in forward-reaching tests (SMD = 2.07; 95% CI = 0.42–3.72; *P* = 0.01). Forward flexion and trunk rotation might be useful strategies to compensate for muscle weakness during transfers and wheelchair propulsion. SCI participants showed reduced trunk displacement during reaching compared to controls, indicating impaired trunk control and sitting balance. Transfer and wheeling trunk strategies vary based on muscle function. Further research on trunk kinematics is needed to guide rehabilitation tailored to individual abilities.

**Supplementary Information:**

The online version contains supplementary material available at 10.1038/s41598-025-06765-5.

## Introduction

Globally, more than 15 million people live with spinal cord injury (SCI)^1^. A significant proportion of persons living with spinal cord injury (PwSCI) rely on wheelchairs for daily mobility, with prevalence rates ranging from 42.9–90.7%^2,3^. For many, navigating the world occurs primarily from a seated position and involves essential activities such as transferring to and from wheelchairs, wheeling for commuting, and interacting with their environment, including reaching for objects.

Trunk movement plays a crucial role in performing seated daily activities^4^ and is closely linked to balance during dynamic tasks^5^. Among PwSCI, improving trunk stability has been identified as a top rehabilitation priority^6^with its decrease associated with reduced mobility independence^7^. Additionally, sitting dynamic stability is often compromised in this population; for example, 69% of wheelchair users report experiencing falls annually^8^. These falls frequently occur during transfers, while wheeling over uneven surfaces, or when reaching for objects^9^.

Given these challenges, assessing trunk movement and control is essential for the population with SCI. Common clinical assessments, such as the Trunk Control Test^10^and Function in Sitting Test^11^are widely used to evaluate trunk function following SCI. While these tools are valuable, kinematic evaluations offer a more objective and precise analysis of movement^12^. Additionally, the degree of independence during transfers and wheeling propulsion varies widely among PwSCI. This variability is influenced by non-modifiable factors such as the extent and level of injury, and modifiable factors such as access to effective training and techniques^13,14^. Rehabilitation techniques must be tailored and adapted to the individual’s specific characteristics, including their muscle function and sometimes compromised sensory system making it challenging to develop optimal movement strategies based on internal sensory feedback^15^. As a result, providing clear and precise instructions for these techniques is critical for enhancing functional independence.

Current guidelines^16^textbooks^17^and assessment tools^18^ offer principles for movement and safety during functional activities for PwSCI. However, much of this information lacks quantitative data on movement, such as degrees of motion. These data aid physiotherapists in identifying specific movement challenges or adaptation techniques used by PwSCI and offer objective metrics to inform tailored rehabilitation strategies. Additionally, while some resources reference trunk movements, they often place greater emphasis on the movement or positioning of the extremities. This focus may overlook the importance of trunk movement in functional activities. For example, increased trunk range of motion and trunk angular velocity have a stronger correlation with the propulsion speed of wheelchairs than upper limb joint movements^7^. Given the evidence, it is crucial to incorporate detailed guidance on optimal trunk movement into current guidelines.

Furthermore, previous reviews have primarily focused on the upper extremities^13,19^provided general descriptions of seated functional tasks^20,21^or examined general neurological populations with flaccid trunk control rather than SCI^22^.

Therefore, the research question for this systematic review was: what are the differences in trunk kinematics between PwSCI and non-injured individuals during sitting-based daily activities?

## Methods

### Design

This systematic review was conducted following the guidelines outlined in the Preferred Reporting Items for Systematic Reviews and Meta-Analyses (PRISMA) statement^23^ (Supplementary Material 1). The review protocol has been prospectively registered in the PROSPERO database under registration number [CRD42024519614]. An amendment was made in PROSPERO to refine the review (24/01/2025), focusing on sitting-based functional tasks rather than functional tasks in general.

### Identification and selection of trials

We conducted a systematic search, including published articles from inception up until 22nd March 2024, with no date restrictions. The following electronic databases were included: MEDLINE (OVID Interface), EMBASE (OVID interface), CINAHL-PLUS (EBSCO interface) and Web of Science. Search terms were agreed by all the authors. The search terms and their synonyms were combined using the Boolean operators “OR” and “AND” and a combination of MeSH and non-MeSH terms were also used. The search terms included spinal cord injury, paraplegia, tetraplegia, movement, kinematics, motion, trunk, torso, and pelvis. A detailed description of the search strategy is provided in Supplementary Material 2. Two independent reviewers (RH and ZA) screened the titles and abstracts of the retrieved articles, assessing them against predetermined criteria (see Table 1). Full-text reviews were then conducted by RH and SYC for eligible studies, supplemented by hand-searching and literature reference lists of included studies to ensure comprehensive coverage. Any disagreement between the two researchers was resolved via discussion with a third researcher (SYC or ZA).

**Table 1:** Inclusion and exclusion criteria.

Criteria	Inclusion criteria	Exclusion criteria
Population	Adults (age ≥ 18 years), Persons living with SCI (PwSCI)	Participants < 18 years
Intervention	Sitting-based functional tasks	Any other type of functional task
Comparison	PwSCI versus non-injured controls	Persons without SCI
Outcome	Trunk kinematics during sitting-based tasks including reaching, independent transfer and wheelchair propulsion.	Trunk kinematics during any other type of non-sitting functional tasks
Study design	Randomised controlled trials, non-randomised controlled trials, observational (both cohort and case-control) studies, cross-sectional studies, case series studies	Case reports, editorials, reviews commentaries, conference abstracts, other systematic reviews
Publication characteristics	Peer-reviewed journal articles, grey literature	Non-peer-reviewed publications, conference posters or abstracts

### Risk of bias assessment

The assessment of bias encountered in the included studies was evaluated using the Joanna Briggs Institute (JBI) critical appraisal tools^24,25^ which provides a structured framework for assessing diverse biases in various study designs. Two independent reviewers (RH and RYW) conducted the bias assessments. The appropriate JBI checklists were applied based on the study design, including those for cross-sectional studies, quasi-experimental studies, and case-series studies. A third reviewer (SYC) was planned to be consulted in instances of disagreement between the two reviewers to resolve discrepancies and determine the final appraisal score, however, the this was not necessary as the two reviewers were able to reach a consensus.

### Data collection and analysis

In addition to the data mentioned above, data extracted from the included studies were also captured on predesigned Microsoft Excel forms and included: first author, year of publication, country of origin, participant characteristics, sample size, task, setting, kinematic equipment and marker placement, software, as well as trunk kinematic outcomes. If the studies did not report mean values and standard deviations, these were extracted from figures using online software (Graphreader.com). Data extraction was performed independently by two reviewers (RH and RYW), and the results were cross verified using a standardized data collection form to ensure accuracy and consistency. For articles meeting the inclusion criteria but lacking essential quantitative data, corresponding authors were contacted for additional information and given six weeks to respond, with a reminder sent at 4 weeks. We contacted four authors, one author sent additional data, two responded within the time but could not provide the data whilst one author did not respond within the six weeks’ timeframe.

Exercise activities were not considered as functional tasks and were therefore excluded. For interventional studies, data collected prior to the application of the intervention was used in this review. Where quantitative data was provided in two or more studies involving the same task (e.g., reaching) and provided the same trunk kinematic measurement (e.g., trunk displacement), a meta-analysis of the pooled data was performed. This analysis utilized Review Manager (RevMan) version 5.4 (Cochrane Informatics, London, UK), with statistical significance defined as a P-value < 0.05. Continuous variables were compared using the standardized mean difference (SMD), and 95% confidence intervals (CIs) were reported for all estimates. Effect sizes are categorized as follows: below 0.3 is small, above 0.5 is moderate, and above 0.8 is large^26^. Heterogeneity among the studies was assessed using the I² statistic to quantify variability. Given the small number of studies included, all pooled data were analysed using a random-effects model to account for potential variability across studies.

A meta-analysis was only possible to analyse reaching performance but not possible for independent transfers and wheelchair propulsion due to lack of quantitative data. For these studies, a narrative synthesis of the data was conducted. Narrative summaries and numerical descriptions were used to present the demographic data and study characteristics. All data were presented by grouping studies into the following tasks: (1) reaching tasks, (2) transfer, and (3) wheelchair propulsion.

## Results

### Study selection

**Fig. 1:**
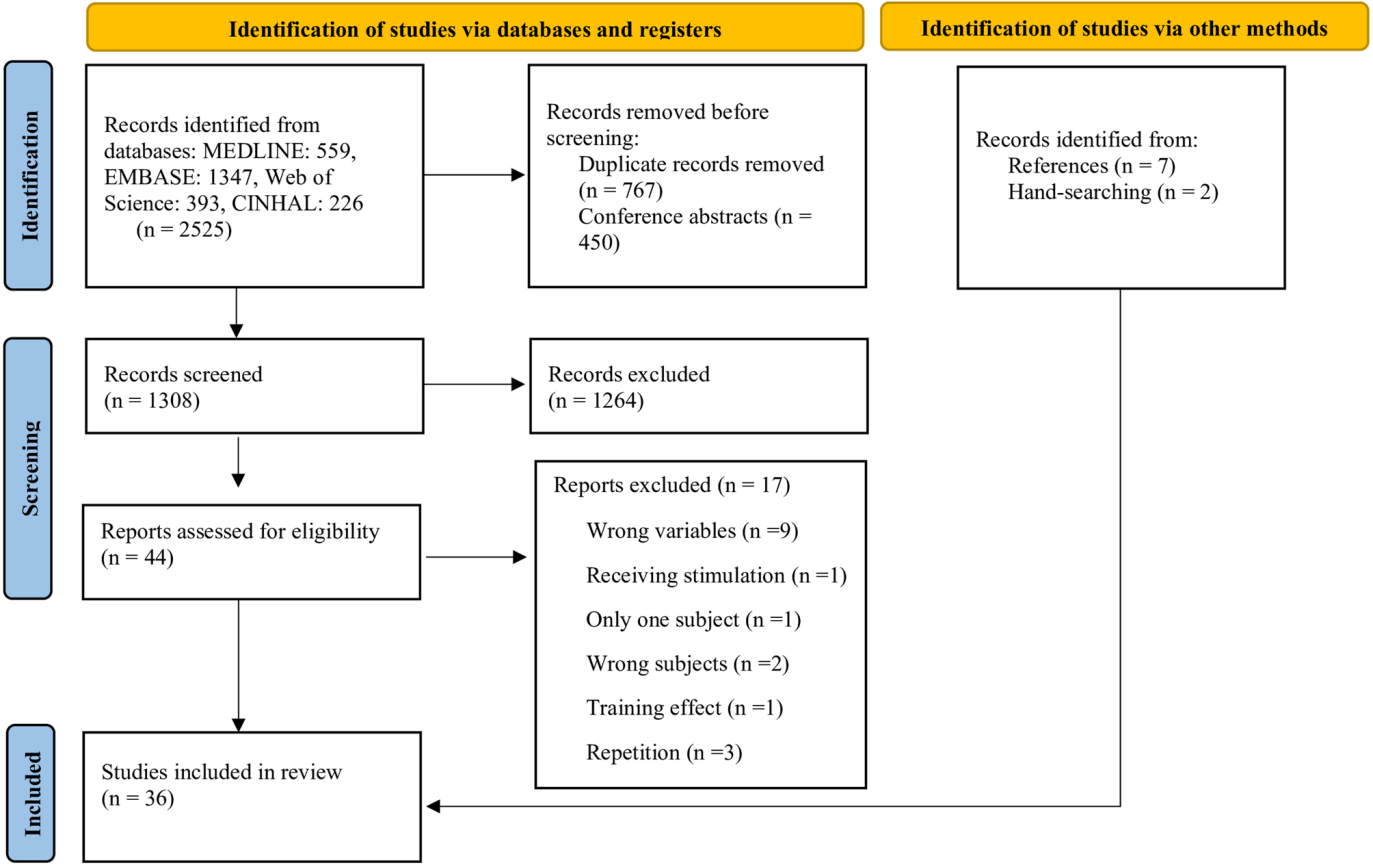
PRISMA flow diagram.

There were a total of 2525 articles eligible in the electronic searching process and after removing duplicates, 1308 articles were screened by titles and abstracts, and 44 were selected for full-text review. During the full-text review process, 27 studies were included and 17 were excluded due to various reasons including: wrong variables (*n* = 9), study repetition (*n* = 3), wrong population (*n* = 2), receiving electrical stimulation (*n* = 1), case-report (*n* = 1), and training effect (*n* = 1). An additional 9 articles were included from hand-searching and literature references. A total of 36 studies were included in this systematic review (Fig. 1).

### Characteristics of the studies

Thirteen studies were performed in the USA^27–40^seven in Canada^41–47^three in Sweden^48–50^three in Japan^51–53^three in the UK^54–56^two in Brazil^57,58^two in Australia^59,60^one in China^61^one in the Netherlands^62^and one in Spain^63^. The total number of participants analysed between all included studies were 444 with a mean age of 39 ± 8 years, 361 were males and the neurological levels of injury ranged from C4 to L2, with the American Spinal Injury Association Impairment Scale (AIS) of A/B (*n* = 272) and C/D (*n* = 109) classifications. The time since injury ranged from six months to 21 years. A summary of the characteristics of the included studies is provided in Table 2. For more detailed characteristics of the studies, see Supplementary Materials 3.

**Table 2:** Summary of characteristics of the included studies.

Study	Origin	Number of participants SCI: Control	Age (yrs)	Level of injury	Time since injury (yrs)	Task	Kinematic method
Reft and Hasan^35^	USA	5:5	26.4 (range 23 to 30)	1 C, 4T (C7-T4)	3–8	Forward reaching; close and far target	3D Marker-based motion capture (Selspot)
Kukke et al.^33^	USA	4:0	35.0 (9)	1 C, 3T (C8 -T8)	3-17.5	Multidirectional Reaching; maximum	3D Marker-based motion capture (VICON)
Kim^30,31^	USA	10:11	39.0 (13.7)	T4-L4	15.9 (9.1)	Multidirectional Reaching; maximum	3D Marker-based motion capture (Qualysis and Flock of Birds)
Field-Fote et al.^28^	USA	32:10	44.9 (11.2)	23Tetraplegia 9Paraplegia	5.1(6.0)	Multidirectional Reaching; maximum	3D Marker-based motion capture (Centennial) and digital video camera (JVC)
de Abreu et al.^58^	Brazil	11:6	30.7 (3.4)	10T,1 L (T2-L1)	2–17	Forward reaching; close and far targets	3D Marker-based motion capture (Polhemus)
Triolo et al.^37^	USA	8:0	46	5T, 3 C (C5-T10)	11.5 (6.9)	Forward reaching; low and high target	3D Marker-based motion capture (VICON)
Rath et al.^34^	USA	8:0	29.4 (7.2)	2 C, 6T (C4-T9)	7.2 (3.1)	Multidirectional Reaching	3D Marker-less motion capture (Xbox One Kinect)
Chiou et al.^56^	United Kingdom	22:16	51.4 (18.1)	9 C, 13T (C2-T10)	6.28 (1–32)	Forward reaching; maximum	3D Marker-based motion capture (VICON)
Castillo-Escario^63^	Spain	24:24	41 (16)	15 C, 9T (C4-T12)	5.7 (4.92) months	Forward reaching; far target	Accelerometer Smartphone (Samsung Galaxy S5)
Janssen-Potten^62^	Netherlands	20:10	High SCI: 32.2 (9.0) Low SCI: 41.5 (11.3)	20T (10 High (level T2–8); 10 Low level T9–12)	> 6months	Forward reaching; far target	3D Marker-based motion capture (Optoelectronic)
Tharu et al.^61^	China	5:0	42 (13.7)	5 C	9.3 (7.4)	Multidirectional Reaching; maximum	3D Marker-based motion capture (VICON)
van Helden et al.^55^	United Kingdom	11:0	57.7 (11.2)	8 C, 3T (C3–T12)	9.1(16.7)	Multidirectional Reaching; maximum	3D Marker-based motion capture (BTS)
Perry et al.^40^	USA	12:0	31.0 (19.8–50.9)	(T8-L1)	8.3 (1.3–20.9)	Sitting pivot transfer Even surface	2D Marker-less motion capture (manual)
Allison et al.^59^	Australia	10:0	30.7 (6.1)	2T 8 C (C5-T10)	7.8 (4.3)	Lateral long-sitting transfer Even surface	2D Marker-based motion capture
Gagnon et al.^44^	Canada	11:0	High-level: 43.7 (3.6) Low-level: 34.6 (11.3)	High-level (C7-T6) Low-level (T11-L2)	High-level: 19.3 (11.2) Low-level: 12.4( 12.6)	Posterior long-sitting transfer Even surface	3D Marker-based motion capture two-camera video technique.
Gagnon et al.^45^	Canada	10:0	39.2 (9.3)	2 C, 6T, 2 L (C7-L2)	15.1 (11.7)	Posterior long-sitting transfer Even and Higher surface	3D Marker-based motion capture two-camera video technique.
Forslund et al.^48^	Sweden	13:0	42.6 (13.2)	13T (T2-10)	16.8 (2–38)	Sitting pivot transfer Higher surface	3D Marker-based motion capture (BTS)
Tanimoto et al.^53^	Japan	11:0	N/A	2 C 8T 1 L (C7-L1)	N/A	Sitting pivot transfer Even surface	2D Marker-less motion capture (manual)
Gagnon et al.^46^	Canada	10:0	41 (9.3)	10T (T4-T11)	12.32	Sitting pivot transfer Even, Higher, Lower surface	3D Marker-based motion capture (Optotrak)
Alonso et al.^57^	Brazil	12:0	32.5 (10.97)	12T (T2-T12)	7.75 (5.83)	Sitting pivot transfer N/A	3D Marker-based motion capture (Qualisys)
Koontz et al.^32^	USA	5:12	40.2 (13.4)	5 T (T4-T12)	17.3 (10.6)	Sitting pivot transfer Even surface	3D Marker-based motion capture (VICON)
Desroches et al.^43^	Canada	26:0 (15 ABD and 11 NABD)	ABD: 42.9 (12.1) NABD: 43.8 (107)	ABD: 14T,1 L (T9-L1) NABD: 1 C, 10T (C7-T7)	ABD: 10.1 (10.8) NABD: 14.7 (13.6)	Sitting pivot transfer Even surface	3D Marker-based motion capture (Optotrak)
Desroches et al.^42^	Canada	32:0	43.9 (10.4)	2 C, 27T, 3 L (C4-L2)	11.6 (10.8)	Sitting pivot transfer Even surface	3D Marker-based motion capture (Optotrak)
Kataoka et al.^52^	Japan	4:0	40 (5.5)	4 C6	12–20	Lateral short-sitting (slide board) Higher surface	3D Marker-based motion capture (ToMoCo VM)
Kankipati et al.^39^	USA	18	36.8 (10.5)	6 C 12T (C5-T12-L1)	13.7 (7.6)	Sitting pivot transfer Even surface	3D Marker-based motion capture (VICON)
Kataoka et al.^51^	Japan	11 39.6 (8.1) 11:0	11 39.6 (8.1) 11:0	11 C6	20 (6.9) (11–31)	Lateral short-sitting Higher surface	2D Marker-less (manual), digital video camera.
Bednarczyk et al.^41^	Canada	10:0	26–52	T6-L2	Chronic	Wheelchair propulsion Self-chosen speed	3D Marker-based motion capture. (Panasonic)
Schantz et al.^50^	Sweden	7:0	Paraplegia (30) Tetraplegia (34)	3 C 4T Paraplegia (T9-T12) Tetraplegia (C5-C7)	Paraplegia: 21 Tetraplegia: 16	Wheelchair propulsion Self-chosen and higher speed	3D Marker-based motion capture
Yang et al.^38^	USA	11:0	41.9 (9.6)	2 C, 9T (C7-T10)	17.5 (9.0)	Wheelchair propulsion 1.3 m/s	3D Marker-based motion capture (Optotrak)
Triolo et al.^36^	USA	6:0	46.0 (10.8)	2 C, 4T (C6-T10)	8.6 (2.8)	Wheelchair propulsion Self-chosen speed	3D Marker-based motion capture (VICON)
Lalumiere et al.^47^	Canada	15:0	38.0 (10.9)	15T (T2-T12)	9.5 (9.4)	Wheelchair Propulsion Curb	3D Marker-based motion capture (Optotrak)
Julien et al.^29^	USA	7:0	33.0 (10.2)	7 C (C5-7)	N/A	Wheelchair propulsion Self-chosen, lower, and higher speed	3D Marker-based motion capture (HiRes)
Symonds et al.^54^	United Kingdom	7:6	42.7 (13.3)	7 (T5-L1)	8.9 (4.7)	Wheelchair propulsion Incline	XSens MTw inertial measurement system
Armstrong et al.^27^	USA	4:0	48.8	3T, 1 C (C7-T4)	19	Wheelchair Propulsion Rapid turn and collision	3D Marker-based motion capture (VICON)
Lili et al.^49^	Sweden	25:0	58.4 (13.8)	17 C, 8T	17.5(15.4 )	Drinking task	3D Marker-based motion capture (Qualisys)
Harvey et al.^60^	Australia	7:0	32.7 (5.9)	7 C (C5-C6)	8.6 (6.2)	Long-sitting weight relief	3D Marker-based motion capture (COHU)

ABD (abdominal control); NABD (non-abdominal control); N/A (not available).

The tasks performed in these studies included short-sitting and long-sitting independent transfers (hips and knees bent or hips flexed, knees extended)^32,39,40,42–46,48,51–53,57,59^wheelchair propulsion activities^27,29,36,38,41,47,50,54^and various reaching performance tasks^28,30,31,33–35,37,55,56,58,61–63^(Supplementary Materials 3).

### Study methodologies

Of the 36 included studies, 32 (88.9%) were conducted in a laboratory setting^27–39,41−50,53–56,58−63^, while four (11.1%) lacked information on the study environment^40,51,52,57^. Of these studies, 29 (80.6%) used marker-based motion capture to assess trunk kinematics^27–33,35−40,42–50,52,55–62^, five (13.9%) used marker-less video analysis^34,41,51,53^one (2.8%) used an inertial measurement system^54^and one (2.8%) employed a smartphone accelerometer^63^. To quantify the trunk kinematics, 24 (66.7%) studies placed markers/landmarks at the spinal column^27–32,34,36,37,39,42–47,50,53,55,56,58,60–62^nine (25%) at the shoulder/acromion^33,35,38,39,41,48,51,52,59^, seven (19.4%) at the sternum^27,29,39,47,49,57,63^one (2.8%) at an unspecified position in the thorax^54^and one (2.8%) did not specify the position^40^. Regarding kinematic outcomes, trunk angular displacement was measured in 29 (80.6%) articles ^27,29–32,34−48,50–54,59−63^, 12 (33.3%) analysed trunk linear displacement^28,33,35,37,48,49,52,55–59^three (8.3%) assessed speed^30,31,35,57^three (8.3%) measured angular velocities^39,46,63^two (5.6%) evaluated trajectory^30,31,57^and one (2.8%) linear velocity^39^ (Supplementary Materials 3).

### Risk of bias

**Fig. 2:**
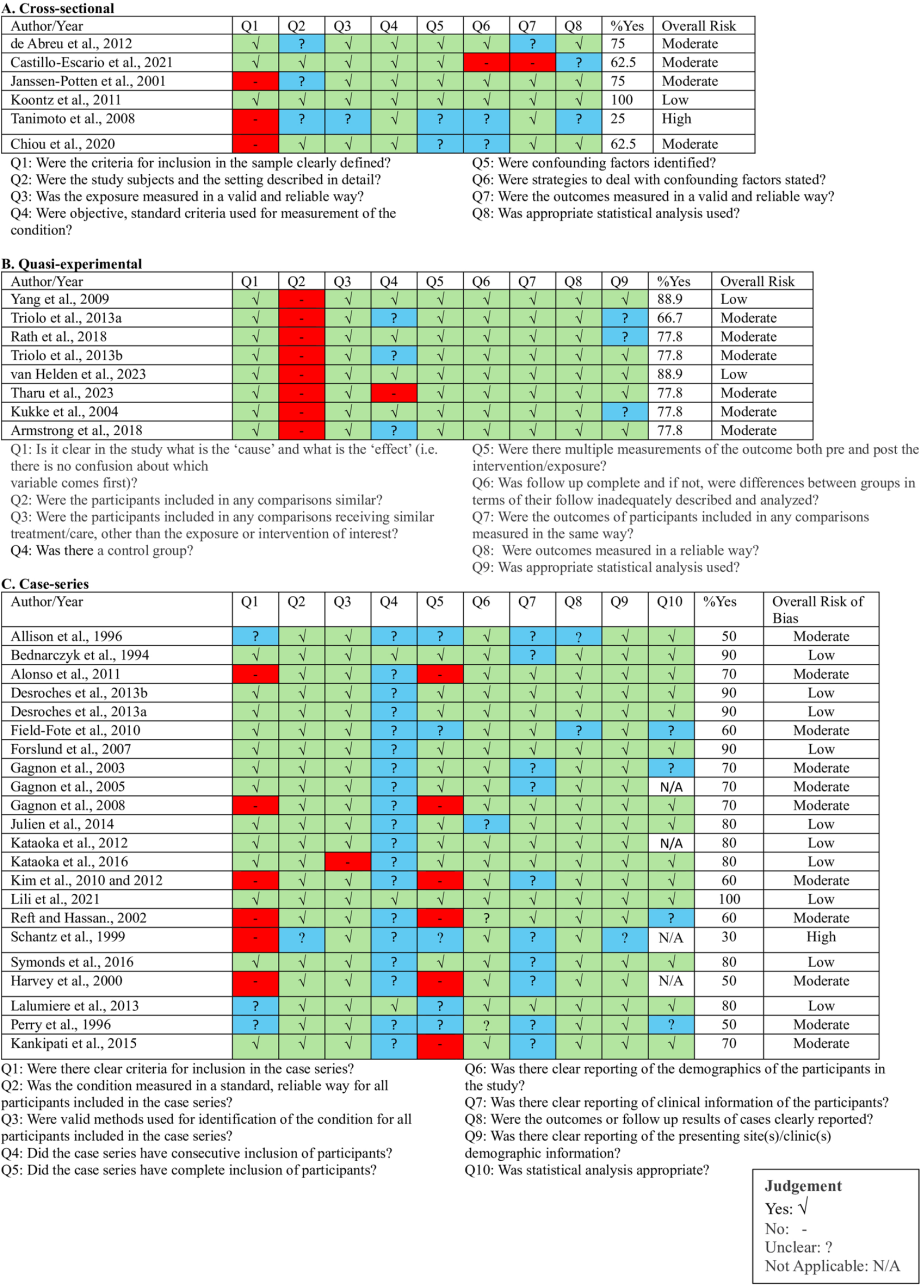
Risk of bias summary in the included studies. Risk of bias in (**A**) cross-sectional studies, (**B**) quasi-experimental studies and (**C**) case-series.

Regarding article quality assessment, 13 (36%) articles presented a low risk of bias^29,32,38,41–43,47−49,51,52,54,55^, 21 (58%) had a moderate risk^27,28,30,31,33–37,39,40,44–46,56−63^, and two (6%) exhibited a high overall risk of bias^50,53^. The scores for each quality criterion and the overall results are detailed in Fig. 2A-C.

Among studies with a moderate and high risk of bias, the most common shortcomings were failing to provide clear inclusion criteria (Q1) and clear reporting of clinical information about participants (Q2 in cross-sectional or Q7 in case series). In cross-sectional studies, each of these issues were presented in 50% (3 studies)^53,56,58,62^ of the total. In case-series studies, 41% (9 studies)^30,31,35,40,46,47,50,57,59,60^ lacked clear inclusion criteria (Q1), and 45% (10 studies)^30,31,39–41,44,45,50,54,59,60^ did not report clinical information of participants (Q7). Furthermore, all case-series studies with moderate to high risk of bias did not report whether participants were consecutively included (Q4), and 83% (10 studies)^28,30,31,35,39,40,46,50,57,59,60^ did not achieve complete inclusion of participants (Q5).

In quasi-experimental studies, none of the included articles provided evidence that participants included in comparisons were similar (Q2). Moderate-risk quasi-experimental studies lost more points due to missing details about control groups (Q4)^27,36,37,61^ or failing to use appropriate statistical methods (Q9)^33,36,37^. These findings highlight the critical need to address these methodological gaps in future research to enhance the reliability and validity of studies in SCI-related trunk kinematics.

### Synthesis of the data from included studies

#### Reaching performance

Compared to non-injured controls during a forward-reaching task, PwSCI demonstrated decreased trunk forward movement, including both linear and angular displacement, and forward pelvic tilt^35,58,62^. Additionally, PwSCI had significantly greater trunk deviations from a straight path, characterized by motion direction, and path curvature index^35^. Moreover, PwSCI required more time to reach targets and displayed slower forward trunk angular velocity compared to non-injured controls^58,63^. A summary of the trunk kinematic results during forward reaching in SCI compared to non-SCI is presented in Supplementary Materials 4.

**Fig. 3:**
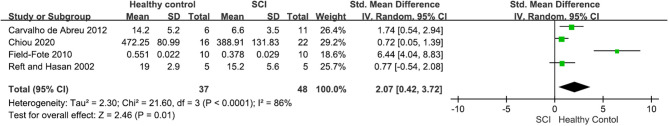
Forest plot to compare comparison of trunk displacement in the forward-reaching test between participants living with spinal cord injury and non-injured controls.

Five articles reporting forward-reaching data were eligible for analysis^28,30,35,56,58^. Although kinematic parameters served as outcome measures across all studies, only trunk linear displacement and trunk peak speed were reported in raw data in at least two studies, enabling analysis. For trunk displacement, four studies were included in the meta-analysis and provided data comparing PwSCI to non-injured controls^28,35,56,58^. Results indicated that non-injured controls demonstrated significantly greater trunk displacement than PwSCI in forward-reaching tests (SMD = 2.07; 95% CI = 0.42–3.72; *P* = 0.01) (Fig. 3). Two studies compared targets at different distances in PwSCI: one set at 110% and 80% of arm length^35^and the other at 90% and 50% of maximal reaching distance^58^. The findings indicated a trend towards greater trunk displacement when reaching for farther targets in PwSCI and non-injured controls. However, a meta-analysis of the data presented by these studies showed that these differences were not statistically significant (SMD = 0.55; 95% CI = -0.16–1.27; *P* = 0.13 for PwSCI and SMD = 1.60; 95% CI = -0.17-3.38; *P* = 0.08 for healthy control) (Supplementary Materials 5 A).

With regards to trunk peak speed, studies have reported conflicting trends, and a meta-analysis showed that the results were not statistically significant (SMD = -1.62; 95% CI = -5.05–1.82; *P* = 0.36) (Supplementary Materials 5B)^31,35^.

#### Independent transfers

When analysing independent transfers, studies identified lateral and posterior long sitting transfers and lateral and pivot transfers during short sitting transfers.

**Fig. 4:**
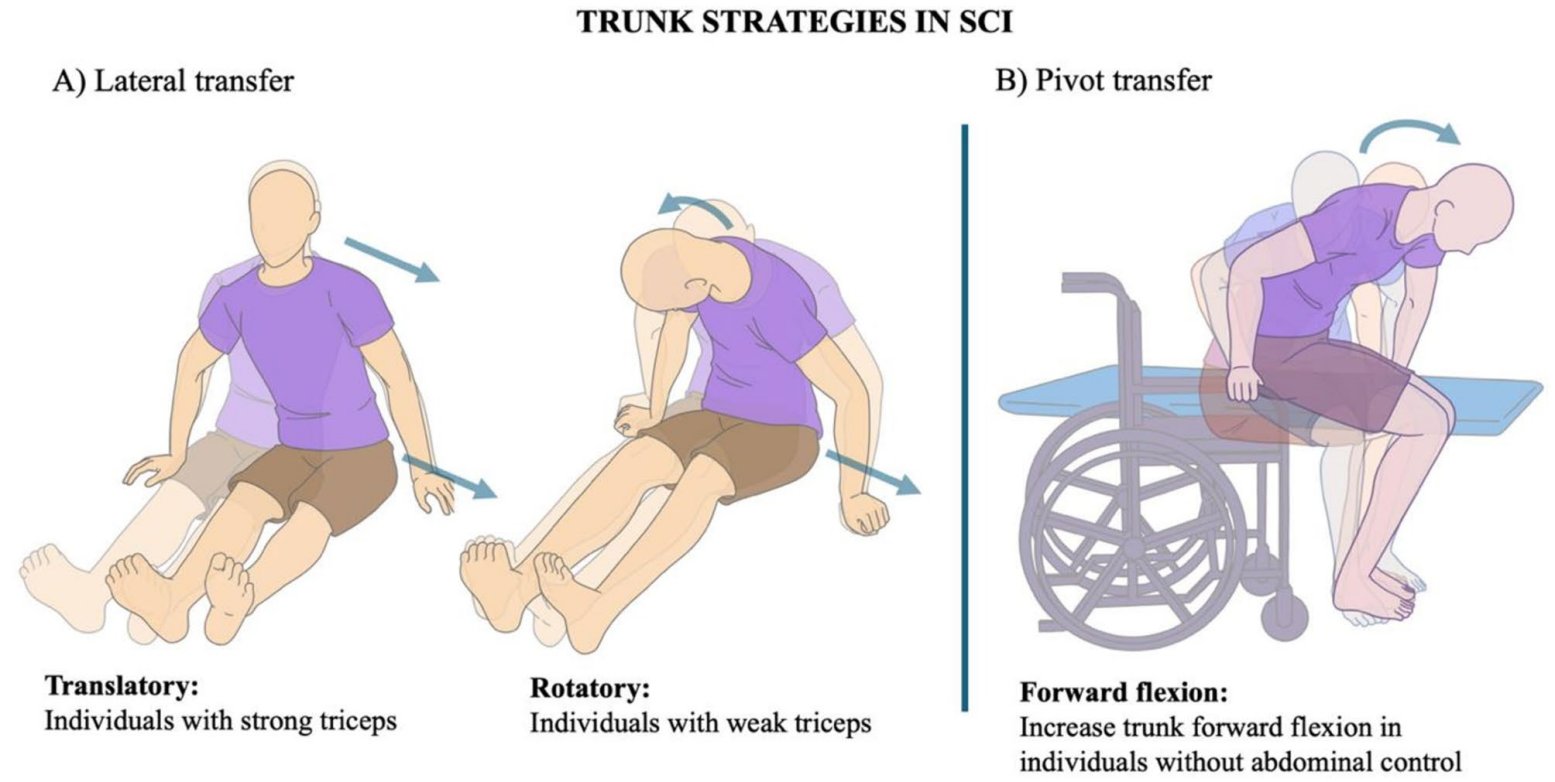
Trunk strategies during transfers. (**A**) Lateral transfer. (adapted from Allison 1993). (**B**) Pivot transfer (based on Desroches 2013b, Perry 1996, Gagnon 2008).

For example, Allison et al.^59^ identified two main strategies used by 10 PwSCI for long-sitting lateral transfers that involved translatory and rotatory strategies. The translatory strategy involved moving the head and pelvis in the same direction, with a strong positive correlation (> 0.70) between their lateral displacements, primarily observed in individuals with thoracic injuries who retain triceps function. Conversely, a rotatory movement where participants moved their head and pelvis in opposite directions, with strong negative correlation (> 0.70) between head and pelvis lateral displacements, was observed in individuals with cervical injuries with weak triceps (See Fig. 4A).

During a posterior transfer on a level surface, participants placed their hands on the floor while lifting their buttocks and performing trunk flexion. Before lifting their buttocks, participants with high-level injuries (C7-T6) exhibited greater forward trunk flexion (19º) compared to those with low-level injuries (T11-L2), who started with an angle of 9.9º. However, both groups reached similar peak trunk flexion (47º) during the lift^44^. Similar strategy, placing both hands on the floor, an increasing the forward trunk flexion to 63.7º was performed to transfer from a low surface to a slightly elevated one, 10 cm higher. This strategy resulted in a higher success rate (70%) compared to the alternate methods (e.g. using one hand on each surface and 59.4º of trunk flexion (success rate: 50%) or elevating both hands and 32º of trunk flexion (success rate: 30%))^45^.

Pivot transfer during short-sitting was performed by 139 participants, and 87.4% of them had thoracic lesions. It consists of three phases: pre-lifting (before buttocks lift from the seat), lifting (buttocks in the air), and post-lifting (buttocks return to the target seat)^32,46,57^. During pre-lift, participants flexed their trunk forward (37.2–57.5º), increasing by 14–18º to peak at 41.6–68º during the lift phase. In post-lift, the trunk gradually extended back to its original position^43,46,53^. The trunk also rotated away from the target^32,43,48^and laterally flexed—initially away (9.4°–22.5°) and then toward the target seat (4.9°–10.5°)^32,40,43^ (See Fig. 4B). Additionally, angular velocity peaked during seat-off and seat-on, with seat-off showing a peak flexion velocity of 50º/s and seat-on a peak extension velocity of 67º/s^46^.

During the pivot transfer, PwSCI exhibited greater trunk angular displacements than those without a SCI: trunk flexion (46.9° vs. 38.2°), rotation (30.6º vs. 18.3º), and lateral flexion (22.6º vs. 9.6º)^32^. Moreover, Desroches et al.^43^ found significantly more flexion in those individuals with SCI lacking abdominal control compared to those with abdominal control (53° vs. 42°).

A lateral transfer technique, involving a transition from a wheelchair to a car seat, was evaluated in two studies that included a total of 15 PwSCI at the C6 level. All participants had challenges in lifting their buttocks due to impaired motor function of the triceps brachii^51,52^. One of the papers specified the use of a sliding board^52^while the other this not specify its use^51^. During transfers, the trunk exhibited repeated rotatory movements, with a strong negative correlation (> 0.70) between head and pelvis lateral displacement, indicating opposite directional movement^52^. This rotation was accompanied by trunk forward flexion (23.6–45.4º)^51,52^.

### Wheelchair propulsion

The wheelchair propulsion cycle consists of two phases: push and recovery^29,38,41,54^. The push phase starts when the hand contacts the pushrim, propelling the wheelchair, while the recovery phase begins when the hand releases the pushrim, allowing the arm to return to the starting position. During the push phase the trunk moves forward to optimize propulsion force, and in the recovery phase the trunk moves backward (total excursion 0-15.7º)^29,38,41,50,54^. Individuals with cervical SCI exhibited greater forward trunk movement compared to those with lower-level lesions during the overground manual wheelchair propulsion^29,36,38,41,54^.

The range of trunk flexion is task-dependent, with significant increases observed during tasks such as faster wheeling speeds, inclines, and curb climbing. For example, trunk flexion increased 5–15° with higher speeds compared to self-pace wheeling^29,50^. Compared to wheeling on a level surface, trunk flexion significantly increases by additional 17º and 21º in inclined surfaces of 6.5% and 12% inclines, respectively. Interestingly, in non-injured novices unaccustomed to wheeling there were not significant changes between level surface and inclines^54^. Trunk flexion also increased with curbs, with maximum flexion recorded at 20º, 32º, and 43º for curb heights of 4 cm, 8 cm, and 12 cm, respectively^47^.

An anterior collision and a high-speed turn task were also examined. Anterior collisions resulted in forward trunk flexion of 16.1–24.7° with a time to return to erect of 0.65–3.94 s, while high-speed turns led to lateral trunk flexion of 1.8–9.5°, with the trunk leaning outward from the turn direction^27^.

## Discussion

This systematic review provides a comprehensive analysis of studies that investigated adaptations of trunk kinematics in PwSCI to allow for effective performance of daily functional activities – reaching, transferring, and manual wheelchair propulsion. Our meta-analysis showed a significant reduction in trunk displacement in PwSCI compared to non-injured controls, suggesting limited control of trunk movement and a reduction in sitting balance post-SCI^28^. Furthermore, our results reveal the details of trunk movement for a successful transfer and manual wheelchair propulsion, essential skills for independence after SCI. Moreover, effective use of trunk rotation allowed individuals with weak triceps brachii to transfer independently.

### Forward reaching

To our knowledge, this is the first meta-analysis to focus specifically on trunk kinematics during forward-reaching tasks in PwSCI. The analysis revealed that PwSCI exhibited significantly less trunk displacement compared to the controls during forward reaching. This trend was consistent across studies, regardless of whether a maximal reaching test or a reaching test with fixed distance was used^28,35,56,58^.

During forward reaching, non-injured individuals coordinate movements of the arms and the trunk, transferring load from the buttocks to the lower limbs, and shifting the centre of pressure (CoP) forward. This requires activation of abdominal, back, and lower limb muscles to maintain balance. To reach further, a greater forward shift of CoP, accompanied by greater muscle activity, is necessary^64,65^. Therefore, individuals with impaired motor function of the trunk and lower extremities often have less CoP excursions during forward reaching^28,64^. CoP excursions have been shown to strongly correlate with maximum trunk displacement in people living with SCI^28^. Therefore, a decrease in trunk displacement may indicate a reduction in the ability to shift the body weight forward without losing balance.

Additionally, to reach for a fixed target, PwSCI compensate with the arms, reduce trunk movement and tilt their pelvis posteriorly, forcing the upper limbs to overextend^31,62,63^. This might increase overextension and overuse of the upper limb, potentially elevating the risk of shoulder overload—a common injury among PwSCI. Our findings underscore the importance of trunk and upper limb dynamics in preventing shoulder injuries.

### Use of trunk forward flexion during independent transfers and manual wheelchair propulsion

Previous research indicates that individuals without abdominal control exhibit approximately 30% greater trunk forward flexion during pivot transfers compared to those with abdominal control^43^. A possible explanation is that a more flexed posture requires less trunk muscle activation^66^ and increases the distance between the CoP and the base of support compared to more upright postures^42^. Increasing forward flexion may facilitate effective weight-shifting from the buttocks to the ground, whilst lowering the centre of mass aiding lift-off and body pivoting^40,48,53^. Interestingly, this strategy may reduce the load in the trailing arm, which typically bears more weight during transfers^39,43^. This supports the theory that increased forward flexion may protect the shoulder joint by engaging the latissimus dorsi and pectoralis major, and reducing the vertical distance between the buttocks and shoulder, alleviating the strain on the joint^40,42,67^.

During wheelchair propulsion greater forward trunk movement was seen in individuals with cervical lesions compared to those with lower-level lesions^29,36,38,41,54^. Similar results were reported during manual wheelchair propulsion on a stationary ergometer where individuals with a C6 SCI increased trunk forward flexion by ~ 5º compared to those with a paraplegia^68^suggesting a compensatory strategy to address muscle weakness. Additionally, when greater push forces are required, wheelchair users tend to increase trunk flexion. For instance, studies have shown that trunk forward flexion increases as the slope of a ramp or the height of curbs rises^36,47,69^. Therefore, increasing trunk flexion may counteract backward forces during wheelchair propulsion and tipping forces experienced on inclines, in addition to improve force generation by optimizing upper-extremity biomechanics^70^. However, its role in shoulder overuse injuries remains unclear^29^.

### Use of trunk rotation for compensation of the impaired upper-limb function during independent transfers

Additional degrees of trunk rotation are observed during pivot transfers in PwSCI compared to non-injured controls^32^. The findings from this review revealed that rotatory trunk movement patterns were also used in other types of transfers by individuals with weak triceps brachii. For instance, during lateral transfers, individuals with weak triceps brachii relied on rotatory trunk movements rather than the translatory movements typically seen in those with strong triceps brachii^52,59^. This occurs because weak triceps brachii makes elbow extension challenging, prompting the use of a rotational technique that leverages trunk angular momentum to lift and reposition the pelvis. These findings suggest that rotatory trunk movements play a crucial role in enabling PwSCI, particularly those with weak triceps brachii, to perform effective and independent transfers.

### Limitations

The are several considerations for our review. Firstly, all studies included in the meta-analysis were judged to possess a moderate risk of bias, potentially affecting the quality to estimate the pooled effect. Another limitation is that most included studies analysed a small sample of individuals (*n* = 4–32), leading to a higher risk of type II error. Moreover, the degree of heterogeneity was high across the included studies; this prevented us from pooling data and performing additional meta-analysis to draw more definitive conclusions. Finally, the trunk strategies during transfers were observed mainly in individuals with motor-complete SCI, limiting clinical relevance for incomplete injuries.

In conclusion, this review highlights that individuals with SCI experience reduced trunk displacement and altered movement patterns during forward reaching, which may impact on overall body positioning during transfers and increases the risk of upper-limb injury. It also identifies trunk movement strategies that can aid independence in transfers and wheelchair propulsion. These findings are highly relevant to physiotherapy practice, enhancing understanding of trunk control and strategies to improve independence. Applying this knowledge can positively influence tailored rehabilitation, improving the lives of individuals with SCI. Ultimately, this review emphasizes the importance of trunk rehabilitation for efficient daily living activities, guiding clinicians in planning effective, personalized physiotherapy for transfers and wheelchair propulsion.

## Electronic supplementary material

Below is the link to the electronic supplementary material.

Supplementary Material 1

## Data Availability

All data generated or analysed during this study are included in this published article (and its Supplementary Information files). Additional requests should be addressed to the corresponding author.
