# Supplementary material for: Trunk kinematics during seated functional activities in individuals with spinal cord injury: a systematic review and meta-analysis
Source: Sci Rep. 2025 Jul 1;15:22276. doi: 10.1038/s41598-025-06765-5 (PMC12219815; doi:10.1038/s41598-025-06765-5)
Supplement: Supplementary file 1 — Supplementary Material 1 [file 41598_2025_6765_MOESM1_ESM.docx]

| Supplemental Appendix 1: PRISMA checklist | | | **Location where item is reported** |
| --- | --- | --- | --- |
| **TITLE** | | |  |
| Title | 1 | Identify the report as a systematic review. | 1 |
| **ABSTRACT** | | |  |
| Abstract | 2 | See the PRISMA 2020 for Abstracts checklist. | 2 |
| **INTRODUCTION** | | |  |
| Rationale | 3 | Describe the rationale for the review in the context of existing knowledge. | 3,4 |
| Objectives | 4 | Provide an explicit statement of the objective(s) or question(s) the review addresses. | 4 |
| **METHODS** | | |  |
| Eligibility criteria | 5 | Specify the inclusion and exclusion criteria for the review and how studies were grouped for the syntheses. | 4,5 and table1 |
| Information sources | 6 | Specify all databases, registers, websites, organisations, reference lists and other sources searched or consulted to identify studies. Specify the date when each source was last searched or consulted. | 4 |
| Search strategy | 7 | Present the full search strategies for all databases, registers and websites, including any filters and limits used. | Supplemental 2 |
| Selection process | 8 | Specify the methods used to decide whether a study met the inclusion criteria of the review, including how many reviewers screened each record and each report retrieved, whether they worked independently, and if applicable, details of automation tools used in the process. | 4,5 |
| Data collection process | 9 | Specify the methods used to collect data from reports, including how many reviewers collected data from each report, whether they worked independently, any processes for obtaining or confirming data from study investigators, and if applicable, details of automation tools used in the process. | 5,6 |
| Data items | 10a | List and define all outcomes for which data were sought. Specify whether all results that were compatible with each outcome domain in each study were sought (e.g. for all measures, time points, analyses), and if not, the methods used to decide which results to collect. | 5,6 |
|  | 10b | List and define all other variables for which data were sought (e.g. participant and intervention characteristics, funding sources). Describe any assumptions made about any missing or unclear information. | 5,6 |
| Study risk of bias assessment | 11 | Specify the methods used to assess risk of bias in the included studies, including details of the tool(s) used, how many reviewers assessed each study and whether they worked independently, and if applicable, details of automation tools used in the process. | 5 |
| Effect measures | 12 | Specify for each outcome the effect measure(s) (e.g. risk ratio, mean difference) used in the synthesis or presentation of results. | 5,6 |
| Synthesis methods | 13a | Describe the processes used to decide which studies were eligible for each synthesis (e.g. tabulating the study intervention characteristics and comparing against the planned groups for each synthesis (item #5)). | 5,6 |
|  | 13b | Describe any methods required to prepare the data for presentation or synthesis, such as handling of missing summary statistics, or data conversions. | 5,6 |
|  | 13c | Describe any methods used to tabulate or visually display results of individual studies and syntheses. | 5,6 |
|  | 13d | Describe any methods used to synthesize results and provide a rationale for the choice(s). If meta-analysis was performed, describe the model(s), method(s) to identify the presence and extent of statistical heterogeneity, and software package(s) used. | 5,6 |
|  | 13e | Describe any methods used to explore possible causes of heterogeneity among study results (e.g. subgroup analysis, meta-regression). | 5,6 |
|  | 13f | Describe any sensitivity analyses conducted to assess robustness of the synthesized results. | 5,6 |
| Reporting bias assessment | 14 | Describe any methods used to assess risk of bias due to missing results in a synthesis (arising from reporting biases). | 5,6 |
| Certainty assessment | 15 | Describe any methods used to assess certainty (or confidence) in the body of evidence for an outcome. | N/A |
| **RESULTS** | | |  |
| Study selection | 16a | Describe the results of the search and selection process, from the number of records identified in the search to the number of studies included in the review, ideally using a flow diagram. | 6, Figure 1 |
|  | 16b | Cite studies that might appear to meet the inclusion criteria, but which were excluded, and explain why they were excluded. | N/A |
| Study characteristics | 17 | Cite each included study and present its characteristics. | 7,7 Table 2, Supplemental 3 |
| Risk of bias in studies | 18 | Present assessments of risk of bias for each included study. | 7,8 Figure 2 |
| Results of individual studies | 19 | For all outcomes, present, for each study: (a) summary statistics for each group (where appropriate) and (b) an effect estimate and its precision (e.g. confidence/credible interval), ideally using structured tables or plots. | 8-11 |
| Results of syntheses | 20a | For each synthesis, briefly summarise the characteristics and risk of bias among contributing studies. | 8-9 |
|  | 20b | Present results of all statistical syntheses conducted. If meta-analysis was done, present for each the summary estimate and its precision (e.g. confidence/credible interval) and measures of statistical heterogeneity. If comparing groups, describe the direction of the effect. | 8-9, Figure 3, Supplemental 5 |
|  | 20c | Present results of all investigations of possible causes of heterogeneity among study results. | 7-9, Figure 3, Supplemental 5 |
|  | 20d | Present results of all sensitivity analyses conducted to assess the robustness of the synthesized results. | N/A |
| Reporting biases | 21 | Present assessments of risk of bias due to missing results (arising from reporting biases) for each synthesis assessed. | 8-9 |
| Certainty of evidence | 22 | Present assessments of certainty (or confidence) in the body of evidence for each outcome assessed. | N/A |
| **DISCUSSION** | | |  |
| Discussion | 23a | Provide a general interpretation of the results in the context of other evidence. | 11-13 |
|  | 23b | Discuss any limitations of the evidence included in the review. | 13 |
|  | 23c | Discuss any limitations of the review processes used. | 13 |
|  | 23d | Discuss implications of the results for practice, policy, and future research. | 13 |
| **OTHER INFORMATION** | | |  |
| Registration and protocol | 24a | Provide registration information for the review, including register name and registration number, or state that the review was not registered. | 4 |
|  | 24b | Indicate where the review protocol can be accessed, or state that a protocol was not prepared. | 4 |
|  | 24c | Describe and explain any amendments to information provided at registration or in the protocol. | 4 |
| Support | 25 | Describe sources of financial or non-financial support for the review, and the role of the funders or sponsors in the review. | 21 |
| Competing interests | 26 | Declare any competing interests of review authors. | 21 |
| Availability of data, code and other materials | 27 | Report which of the following are publicly available and where they can be found: template data collection forms; data extracted from included studies; data used for all analyses; analytic code; any other materials used in the review. | N/A |

*From:*  Page MJ, McKenzie JE, Bossuyt PM, Boutron I, Hoffmann TC, Mulrow CD, et al. The PRISMA 2020 statement: an updated guideline for reporting systematic reviews. BMJ 2021;372:n71. doi: 10.1136/bmj.n71. This work is licensed under CC BY 4.0. To view a copy of this license, visit <https://creativecommons.org/licenses/by/4.0/>

**Supplementary Material 2.** Systematic Review Search.

**Embase** <1974 to 2024 March 22>

| # | Query | Results from 25 Mar 2024 |
| --- | --- | --- |
| 1 | exp spinal cord injury/ | 93,368 |
| 2 | spinal cord injur*.mp. | 83,004 |
| 3 | 1 or 2 | 103,060 |
| 4 | exp paraplegia/ | 26,734 |
| 5 | exp quadriplegia/ | 21,081 |
| 6 | exp "movement (physiology)"/ | 531,387 |
| 7 | movement*.mp. | 586,342 |
| 8 | 6 or 7 | 917,828 |
| 9 | kinematic*.mp. | 62,961 |
| 10 | exp motion/ | 207,275 |
| 11 | motion.mp. | 353,593 |
| 12 | 10 or 11 | 485,432 |
| 13 | exp trunk/ | 118,126 |
| 14 | trunk.mp. | 105,516 |
| 15 | 13 or 14 | 192,619 |
| 16 | torso.mp. | 9,839 |
| 17 | exp pelvis/ | 131,768 |
| 18 | pelvis.mp. | 191,647 |
| 19 | 17 or 18 | 249,489 |
| 20 | 8 or 9 or 12 | 1,258,911 |
| 21 | 15 or 16 or 19 | 438,938 |
| 22 | 3 or 4 or 5 | 135,051 |
| 23 | 20 and 21 and 22 | 1,347 |

**Ovid MEDLINE**(R) ALL <1946 to March 22, 2024>

| # | Query | Results from 25 Mar 2024 |
| --- | --- | --- |
| 1 | exp Spinal Cord Injuries/ | 56,881 |
| 2 | spinal cord injur*.mp. | 61,011 |
| 3 | 1 or 2 | 72,196 |
| 4 | exp Paraplegia/ | 13,610 |
| 5 | exp Quadriplegia/ | 8,494 |
| 6 | exp Movement/ | 666,554 |
| 7 | movement*.mp. | 616,238 |
| 8 | 6 or 7 | 998,194 |
| 9 | kinematic*.mp. | 48,777 |
| 10 | exp Motion/ | 72,557 |
| 11 | motion.mp. | 299,202 |
| 12 | 10 or 11 | 339,596 |
| 13 | exp Torso/ | 232,254 |
| 14 | torso.mp. | 11,555 |
| 15 | 13 or 14 | 238,252 |
| 16 | trunk.mp. | 66,414 |
| 17 | pelvis.mp. | 75,301 |
| 18 | 3 or 4 or 5 | 86,452 |
| 19 | 8 or 9 or 12 | 1,270,836 |
| 20 | 15 or 16 or 17 | 343,791 |
| 21 | 18 and 19 and 20 | 559 |

CINHAL

| Movement*, motion, kinematic* | 243,405 |
| --- | --- |
| Pelvis, trunk, torso | 36,344 |
| Spinal cord injur*, paraplegia, quadriplegia | 28773 |
| Result | 226 |

WEB OF SCIENCE

TS=(spinal cord injury OR tetraplegia OR paraplegia OR quadriplegia) 102,740

AND

TS=(movement OR motion OR kinematics) 1,969,020

AND

TS=(trunk OR torso OR pelvis) 141,075

Result: 393

**Supplemental Material 3.** Summary table.

| **REACHING** |  |  |  |  |  |  |  |
| --- | --- | --- | --- | --- | --- | --- | --- |
|  | **N SCI**  **Age (years)**  **Sex (m:f)** | **Level of injury**  **AIS** | **Time since injury (years)** | **N Healthy**  **Age (years)**  **Sex (m:f)** | **Task**  **Setting** | **Kinematic Method**  **Landmark**  **Analysis Method** | **Trunk Kinematic Outcomes** |
| Reft and Hasan 2002  USA | 5  25.8 (23- 35)  4:1 | 1C,4T (C7-T4)  1A, 4B | 3 - 8 | 5  26.4 (23 to 30)  5:0 | Forward reaching; close and far target  Laboratory | 3D Marker-based motion capture (Selspot)  Acromion  N/A | -Speed  **-**Path curvature index.  -Linear displacement: flexion  -Motion direction: flexion (0º anterior, 90ºupward, 180º posterior sagittal plane) |
| Kukke et al., 2004  USA | 4  35.0 (9)  3:1 | 1C, 3T  (C8 -T8)  2A, 2B | 3-17.5 | - | Multidirectional Reaching; maximum  Laboratory | 3D Marker-based motion capture (VICON)  Acromion  MATLAB | -Linear displacement: flexion and upward shift  -Workspace volume (3D) |
| Kim 2010 and 2012  USA | 10  39.0 (13.7)  10:0 | T4-L4  A/B | 15.9 (9.1) | 11  27.5 (10.8)  8:3 | Multidirectional Reaching; close and far target  Laboratory | 3D Marker-based motion capture (Qualysis and Flock of Birds)  C7/T1 and L5/S1  N/A | -Angular displacement: Flexion/ Extension, lateral flexion, rotation (angle between the vertical line and the line between L5/S1-C7/T1)  -Trajectory: flexion (C7/T1)  -Speed (C7/T1) |
| Field-Fote et al., 2010  USA | 32  44.9 (11.2)  25:7 | 23 Tetraplegia  9 Paraplegia  25C, 7D | 5.1(6.0) | 10  41.4 (13.6)  6:4 | Multidirectional Reaching; maximum  Laboratory | 3D Marker-based motion capture (Centennial) and digital video camera (JVC)  C7  N/A | -Linear displacement: Flexion/extension and lateral flexion |
| de Abreu et al., 2012  Brazil | 11  30.7 (3.4)  N/A | 10T,1L  (T2-L1)  7A, 1B, 3C | 2-17 | 6  26.33 (2.73)  N/A | Forward reaching; close and far targets  Laboratory | 3D Marker-based motion capture (Polhemus)  C7  N/A | -Linear displacement: flexion  -Time spent to perform flexion/extension |
| Triolo et al., 2013  USA | 8  46  6:2 | 5T, 3C (C5-T10)  3A 3B 2C | 11.5 (6.9) | - | Forward reaching; low and high target  Laboratory | 3D Marker-based motion capture (VICON)  C7 and mid-PSIS  N/A | -Linear displacement: flexion  -Angular displacement: flexion (angle between vertical line and the line from C7-mid PSIS)  Pelvic angle: flexion (angle between mid-PSIS and horizontal) |
| Rath et al., 2018  USA | 8  29.4 (7.2)  7:1 | 2C, 6T (C4-T9)  6 A, 2C | 7.2 (3.1) | - | Multidirectional Reaching  Laboratory | 3D Marker-less motion capture (Xbox One Kinect)  24 segments of which the head, neck,C7, T8, T12, L5 and sacrum (lower trunk), and ASIS  Custom MATLAB code | -Angular displacement: flexion (angle between the horizontal line intersecting the ASIS and line head-ASIS  -Spinal alignment: anterior-posterior, and medio-lateral  -Trunk curvature |
| Chiou et al., 2020  United Kingdom | 22  51.4 (18.1)  13:9 | 9C, 13T (C2-T10)  11C, 11D | 6.28 (1-32)  chronic | 16  29.7 (10.9)  8:0 | Forward reaching; maximum  Laboratory | 3D Marker-based motion capture (VICON)  T1  Vicon Nexus software | -Linear displacement: flexion |
| Castillo-Escario, 2021  Spain | 24  41 (16)  16:8 | 15C, 9T  (C4-T12)  7A, 4B, 9C, 4D | 5.7 (4.92) months | 24  42 *(*14)  16:8 | Forward reaching; far target  Laboratory | Accelerometer Smartphone (Samsung Galaxy S5)  Sternum  MATLAB | -Angular displacement: flexion and lateral deviation  -Angular velocity: flexion |
| Janssen-Potten, 2001  Netherlands | 20  High SCI: 32.2 (9.0)  Low SCI: 41.5 (11.3)  19:1 | 20T (10 High (level T2–8); 10 Low level T9–12)  20A | N/A: >6months | 10  35.3 (8.9)  9:1 | Forward reaching; far target  Laboratory | 3D Marker-based motion capture (Optoelectronic)  PSIS, ASIS  PRIMASYS | -Pelvic angular displacement: flexion (angle initial position of the line PSIS-ASIS and final position) |
| Tharu et al., 2023  China | 5  42 (13.7)  2:3 | 5C  5A | 9.3 (7.4) | - | Multidirectional Reaching; maximum  Laboratory | 3D Marker-based motion capture (VICON)  C7 and S1  MATLAB | Angular displacement: flex/extension, lateral flexion, and rotation (angle between vertical line and line C7-S1) |
| van Helden et al., 2023  United Kingdom | 11  57.7 (11.2)  9:2 | 8C, 3T (C3–T12)  8C, 3D | 9.1(16.7) | - | Multidirectional Reaching; maximum  Laboratory  . | 3D Marker-based motion capture (BTS)  T1  MATLAB | Linear displacement: flexion and lateral flexion |

| **TRANSFERS** | | | | | | |
| --- | --- | --- | --- | --- | --- | --- |
| **Author**  **Country** | **N SCI**  **Age (years)**  **Sex (m:f)** | **Level of injury**  **AIS** | **Time since injury (years)** | **Task**  **WC angle position; Surface level**  **Setting** | **Kinematic Method**  **Landmark**  **Analysis method** | **Trunk Kinematic Outcomes** |
| Perry et al., 1996  USA | 12  31.0 (19.8-50.9)  12:0 | (T8-L1)  Complete | 8.3 (1.3-20.9) | Sitting pivot transfer  Self-selected angle; Even surface  N/A | 2D Marker-less motion capture (manual)  N/A  N/A | -Qualitative angular displacement: flexion, lateral flexion, rotation. |
| Allison et al., 1996  Australia | 10  30.7 (6.1)  10:0 | 2T 8C (C5-T10)  N/A | 7.8 (4.3) | Lateral long-sitting transfer  Even surface  Laboratory | 2D Marker-based motion capture  Acromion, trochanter, iliac crest  N/A | - Qualitative angular displacement: flexion (acromion-trochanter line)  - Linear displacement: lateral flexion(iliac crest) |
| Gagnon et al., 2003  Canada | 11  High-level: 43.7 (3.6)  Low-level: 34.6 (11.3)  11:0 | High-level (C7-T6)  Low-level (T11-L2)  11A/B | High-level: 19.3 (11.2)  Low-level: 12.4( 12.6) | Posterior long-sitting transfer  Even surface  Laboratory | 3D Marker-based motion capture  two-camera video technique.  N/A: 3 non-collinear markers used to define the trunk segment  Custom-made MATLAB | -Angular displacement: flexion |
| Gagnon et al., 2005  Canada | 10  39.2 (9.3)  10:0 | 2C, 6T, 2L (C7-L2)  10 A/B | 15.1 (11.7) | Posterior long-sitting transfer  Even and Higher surface  Laboratory | 3D Marker-based motion capture  two-camera video technique.  N/A: 3 non-collinear markers used to define the trunk segment  Custom-made MATLAB | -Angular displacement: flexion |
| Forslund et al., 2007  Sweden | 13  42.6 (13.2)  7:6 | 13T (T2-10)  11A 2B | 16.8 (2-38) | Sitting pivot transfer  Self-selected angle; Higher surface  Laboratory | 3D Marker-based motion capture (BTS)  Acromion  Axograph | -Angular displacement: rotation (angle between acromion and the anterior-posterior axis)  -Linear displacement: flexion |
| Tanimoto et al., 2008  Japan | 11  N/A  10:1 | 2C 8T 1 L (C7-L1)  N/A | N/A | Sitting pivot transfer  Self-selected angle; Even surface  Laboratory | 2D Marker-less motion capture (manual)  Neck and buttocks  N/A | -Angular displacement: flexion (angle between the vertical axis and line end of the neck-buttocks. |
| Gagnon et al., 2008  Canada | 10  41 (9.3)  10:0 | 10T (T4-T11)  10A | 12.32 | Sitting pivot transfer  90º angle; Even, Higher, Lower surface  Laboratory | 3D Marker-based motion capture (Optotrak)  N/A: 6 markers with custom-made programs  Custom-made MATLAB | -Angular displacement: flexion/extension (angle between the trunk segment and longitudinal axis which is pedicular to the ground)  -Angular velocity: flexion/extension |
| Alonso et al., 2011  Brazil | 12  32.5  (10.97)  12:0 | 12T (T2-T12)  11A 1C | 7.75 (5.83) | Sitting pivot transfer  N/A  N/A | 3D Marker-based motion capture (Qualisys)  Sternum.  Qtrac View, Qtools, MATLAB, Biostatistic 4.0 and Origin. | -Linear displacement: flexion  -Trajectory: flexion  -Index of curvature  -Speed  -Acceleration |
| Koontz et al., 2011  USA | 5  40.2 (13.4)  N/A | 5 T (T4-T12)  A/B | 17.3 (10.6) | Sitting pivot transfer  Self-selected angle; Even surface  Laboratory | 3D Marker-based motion capture (VICON)  N/A: 6 markers with custom-made programs  MATLAB | -Angular displacement: flexion, lateral flexion, rotation |
| Desroches et al., 2013*  Canada | 26 (15 ABD and 11 NABD)  ABD: 42.9 (12.1)  NABD: 43.8 (107)  ABD: 13:2  NABD: 11:0 | ABD:  14T,1L (T9-L1)  NABD: 1C, 10T (C7-T7)  ABD: 13A 2B  NABD: 8A 3B | ABD: 10.1 (10.8)  NABD: 14.7 (13.6) | Sitting pivot transfer  90º angle; Even surface  Laboratory | 3D Marker-based motion capture (Optotrak)  N/A: 6 markers with custom-made programs  N/A | -Angular displacement: flexion/extension, rotation, and lateral flexion (relative motion between the trunk and the pelvis) |
| Desroches et al., 2013  Canada | 32  43.9 (10.4)  30:2 | 2C, 27T, 3L (C4-L2)  19A, 6B, 3C, 4D | 11.6 (10.8) | Sitting pivot transfer  90º angle; Even surface  Laboratory | 3D Marker-based motion capture (Optotrak)  N/A: 6 markers with custom-made programs  N/A | -Angular displacement: flexion/extension (angle between the trunk segment and longitudinal axis which is pedicular to the ground) |
| Kataoka et al., 2012  Japan | 4  40 (5.5)  4:0 | 4 C6  4A | 12-20 | Lateral short-sitting (slide board)  Self-selected angle; Higher surface  N/A | 3D Marker-based motion capture (ToMoCo VM)  Acromion and trochanter.  N/A | -Angular displacement: flexion (angle between the vertical axis and the line from the middle of right and left acromion to the middle of right and left trochanter)  -Linear displacement: lateral flexion. |
| Kankipati et al., 2015  USA | 18  36.8 (10.5)  17:1 | 6C 12T  (C5-T12-L1)  N/A | 13.7 (7.6) | Sitting pivot transfer  Self-selected angle; Even surface  Laboratory | 3D Marker-based motion capture (VICON)  Acromion, xiphoid, C7 and T3  MATLAB | -Angular displacement: flexion (C7-T3 line)  -Angular velocity: flexion and rotation  -Linear velocity: flexion and rotation |
| Kataoka et al., 2016  Japan | 11  39.6 (8.1)  11:0 | 11 C6  11A | 20 (6.9)  (11–31) | Lateral short-sitting  Self-selected angle; Higher surface  N/A | 2D Digital video camera. Marker-less, manual labelling.  Acromion, trochanter, knee  Image-J (NIH) | -Angular displacement: trunk flexion (angle between the vertical axis and the line from the acromion to the trochanter) and hip flexion (angle between the line from the acromion to the trochanter and the line between the trochanter to the knee. |

| **WHEELCHAIR USE** | |  |  |  |  |  |
| --- | --- | --- | --- | --- | --- | --- |
| **Author**  **Country** | **N SCI**  **Age (years)**  **Sex (m:f)** | **Level of injury**  **AIS** | **Time since injury (years)** | **Task**  **Setting** | **Kinematic Method**  **Landmark**  **Analysis method** | **Trunk Kinematic Outcomes** |
| Bednarczyk et al., 1994  Canada | 10  26-52  7:3 | T6-L2  N/A | N/A:Chronic | Wheelchair propulsion  Self-chosen speed  Laboratory | 3D Marker-based Digital video cameras (Panasonic)  Hip and shoulder, C7  BMDP Biomedical Computer | -Angular displacement: flexion (angle between the vertical line from the hip, and the line hip-shoulder) |
| Schantz et al., 1999  Sweden | 7 (4 Paraplegia, 3 Tetraplegia)  Paraplegia (30)  Tetraplegia (34)  Paraplegia 3:1  Tetraplegia 4:0 | 3C 4T  Paraplegia (T9-T12)  Tetraplegia (C5-C7)  N/A | Paraplegia: 21  Tetraplegia: 16 | Wheelchair propulsion  Self-chosen and higher speed  Laboratory | 3D Marker-based motion capture  C7  N/A | -Angular displacement: flexion (the reference line in resting position C7-top dead centre (12 o clock) of the rim and the same line during propulsion. |
| Yang et al., 2009  USA | 11  41.9 (9.6)  9:2 | 2C, 9T (C7-T10)  7A, 3B, 1C | 17.5 (9.0) | Wheelchair propulsion  1.3 m/s  Laboratory | 3D Marker-based motion capture (Optotrak)  Acromion and trochanter  N/A | -Angular displacement: trunk flexion; the angle between a reference line in resting position (acromion to trochanter) and the same line during propulsion. |
| Triolo et al., 2013*  USA | 6  46.0 (10.8)  4:2 | 2C, 4T (C6-T10)  3A 2B 1C | 8.6 (2.8) | Wheelchair propulsion  Self-chosen speed  Laboratory | 3D Marker-based motion capture (VICON)  C7 and T6  N/A | -Angular displacement: flexion (angle between the line C7-T6 with the vertical axis) |
| Lalumiere et al., 2013  Canada | 15  38.0 (10.9)  14:1 | 15T (T2-T12)  13A 1B 1C | 9.5 (9.4) | Wheelchair  Propulsion  Curb  Laboratory | 3D Marker-based motion capture (Optotrak)  C7, sternal notch, T8, xiphoid  Custom-made MATLAB | -Angular displacement: trunk flexion/extension (line created with the midpoint between T8 and the xiphoid to the midpoint between C7 and the sternal notch, with respect to the vertical axis. |
| Julien et al., 2014  USA | 7  33.0 (10.2)  5:2 | 7 C (C5-7)  3A, 2B, 1C, 1D | N/A | Wheelchair propulsion  Self-chosen, lower, and higher speed  Laboratory | 3D Marker-based motion capture (HiRes)  Sternum, C4, T4, and T7  MATLAB | -Angular displacement: flexion, lateral flexion, rotation. |
| Symonds et al, 2016  United Kingdom | 7  42.7 (13.3)  7:0 | 7 (T5-L1)  N/A | 8.9 (4.7) | Wheelchair propulsion  Incline  Laboratory | XSens MTw inertial measurement system  Thorax  MATLAB | -Angular displacement: flexion |
| Armstrong et al., 2018  USA | 4  48.8  2:2 | 3T, 1C  (C7-T4)  2A, 2B | 19 | Wheelchair  Propulsion  Rapid turn and collision  Laboratory | 3D Marker-based motion capture (VICON)  Sternum and C7  MATLAB | -Angular displacement: flexion, lateral flexion  -Average return time to erect |
| **OTHERS** |  |  |  |  |  |  |
| Lili et al., 2021  Sweden | 25  58.4 (13.8)  18:7 | 17C, 8T  10A, 4B, 3C, 8 D | 17.5(15.4 ) | Drinking task  Laboratory | 3D Marker-based motion capture (Qualisys)  Sternum  MATLAB | Linear displacement: flexion |
| Harvey et al., 2000  Australia | 7  32.7 (5.9)  7:0 | 7 C  (C5-C6)  7A/B | 8.6 (6.2) | Long-sitting weight relieve  Laboratory | 3D Marker-based motion capture (COHU)  Acromion, C7, T4, T12, sternum, and sacrum  Amass software | -Descriptive flexion |

Abbreviations: ABD (abdominal control);NABD (non-abdominal control);N/A (not available)

**Supplementary Materials 4.** Trunk kinematics during forward reaching in SCI in comparison with non-SCI.

| **Parameter** | **Main results** | **References** |
| --- | --- | --- |
| Linear Displacement (cm) | SCI < Non-SCI | Reft and Hasan 2002*; Field-Fote 2010^Ns^; de Abreu et al., 2012*  Chiou et al., 2020 ^Ns^ |
| Angular displacement (º) | Thoracic SCI < Non-SCI  < Cervical SCI | Kim 2010 and 2012; Castillo Escario 2021* |
| Peak velocity (cm/s) | Conflicting | Kim 2010 and 2012*; Reft and Hasan 2002 |
| Motion direction (º) | SCI > Non-SCI | Reft and Hasan 2002* |
| Forward pelvic tilt (º) | SCI < Non-SCI | Janssen-Potten, 2021* |
| Trajectory  (cm) | SCI > Non-SCI | Kim 2010 and 2012 |
| Movement time (s) | SCI > Non-SCI | Abreu et al., 2012* |
| Angular velocity (º/s) | SCI < Non-SCI | Castillo Escario 2021* |
| Path curvature index | SCI > Non-SCI | Reft and Hasan 2002* |
| Lateral deviation (º) | Conflicting | Castillo Escario 2021*; Kim 2010 and 2012 |
| Rotation deviation  (º) | SCI > Non-SCI | Kim 2010 |

^Ns^ No statistical analysis; * Significant

Conflicting: trends in opposite directions.

**Supplementary Material 5.** Additional Forest plot


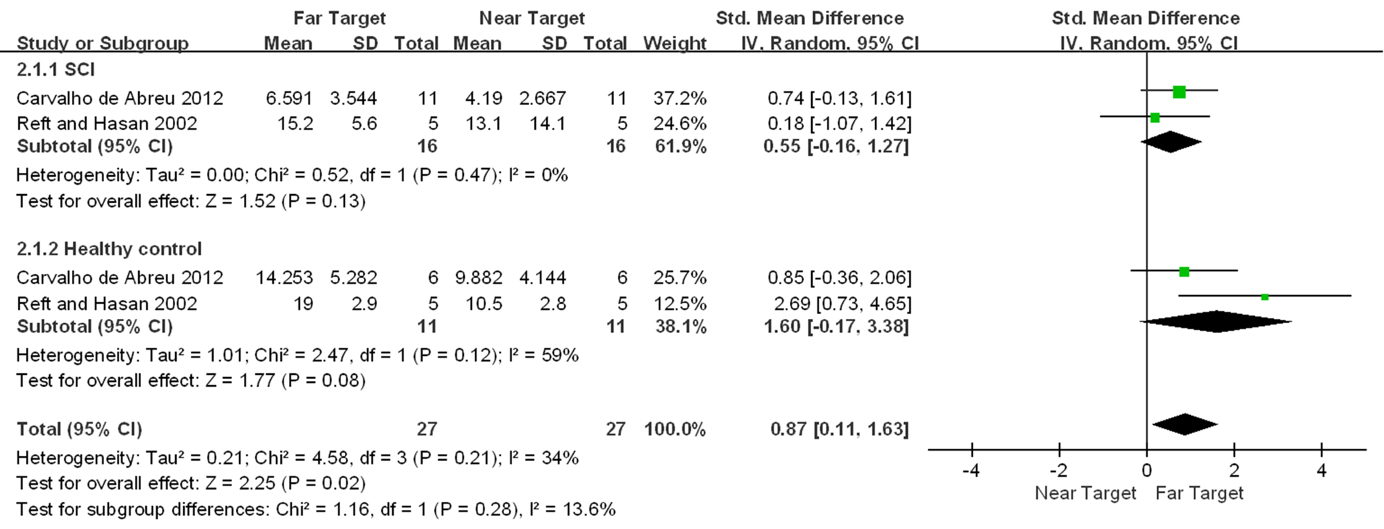


A) Forest plot: Comparison of Trunk Displacement for Far vs. Near Targets in Forward-Reaching Test. Individuals with spinal cord injury and healthy controls.


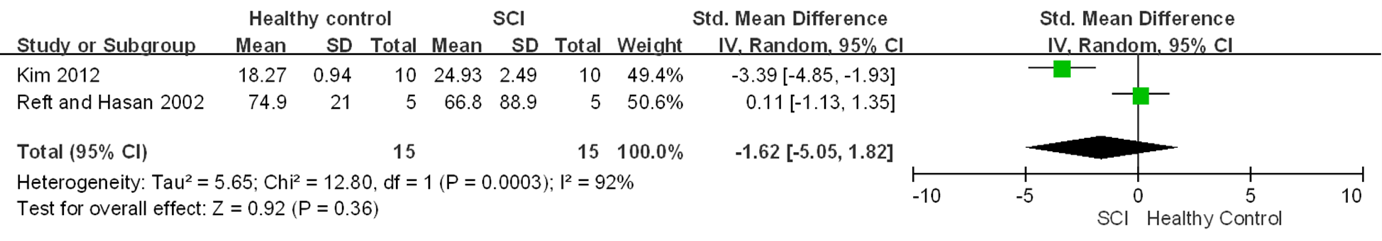


B) Forest plot: Comparison of Trunk Peak Speed in Forward-Reaching Test between SCI Participants and Healthy Controls
